# Complete genome sequences of the biocontrol strains *Bacillus velezensis* MRL14-522 and MRL14-108

**DOI:** 10.1128/mra.00462-26

**Published:** 2026-08-21

**Authors:** Margot Grimonpont, Estelle Turc, Noadya Monnier, Marc Bardin

**Affiliations:** 1 INRAE, Pathologie Végétale, Montfavet, France; 2 Groupe Elephant Vert France, Serris, France; California State University San Marcos, San Marcos, California, USA

**Keywords:** genomes, *Bacillus velezensis*, biological control

## Abstract

*Bacillus velezensis* strains MRL14-522 and MRL14-108 are plant-associated isolates with demonstrated biocontrol activity. This study presents the complete genome sequence of both strains. They consist of a circular chromosome (3,998,688 and 4,057,033 bp, respectively) and an intact prophage (NC_028969 and NC_029104, respectively).

## ANNOUNCEMENT

Most commercially available *Bacillus* strains used as biocontrol agents against plant pathogens belong to the species *Bacillus velezensis*, even though they are frequently registered under the names *Bacillus subtilis* or *Bacillus amyloliquefaciens* (1–4). Strains MR14-522 and MRL14-108 were isolated in 2014 from *Primula vulgaris* seeds and *Sclerotinia sclerotiorum* sclerotia collected in infested soil in Avignon, France (43°56′45.762″N, 4°51′54.768″E), respectively. Strains were obtained on 10% tryptic soy agar, purified, and stored in 20% glycerol-phosphate buffer at −20°C or −80°C. Their biocontrol activity and their ability to synthesize secondary antifungal metabolites were recently demonstrated (5).

The strains were cultured in 50 mL of Lysogeny Broth at 21°C and 200 rpm for 24 h. Genomic DNA was extracted using the Qiagen Genomic DNA Buffer Set (reference 19060) and Genomic-tip 100/G Kit (reference 10243).

Whole-genome sequencing was performed at the Institute for Integrative Biology of the Cell platform (Gif-sur-Yvette, France) using Illumina and Oxford Nanopore technologies with libraries enriched for fragments >3 kb, using the SQK-LSK109 long fragment buffer.

Oxford Nanopore sequencing was performed on a GridION platform using an R9.4.1 flow cell. Basecalling was performed using Guppy v4.0.11 included in MinKNOW v20.06.09 (Oxford Nanopore Technologies). A total of 471,099 raw reads (N50, 28,338 bp) and 485,440 raw reads (N50, 24,129 bp) were obtained for strains MRL14-522 and MRL14-108, respectively. Adapter sequences were trimmed using Porechop v0.2.4 (https://github.com/rrwick/Porechop).

Illumina sequencing libraries were prepared using the TruSeq genomic library preparation kit. Libraries were sequenced on a NextSeq500 platform using single-end reads (86 bp), generating approximately 4,078,088 reads for MRL14-522 and 4,883,881 reads for MRL14-108 after quality filtering. Reads were quality-filtered using FastQC v0.11.5 (6) and adapter trimming performed using Cutadapt v1.15 (7).

*De novo* assembly was performed using Canu 1.7 (8). Circular contig assemblies were polished with Nanopolish v0.13.2 (9), followed by iterative correction of single-base errors and indels with Illumina reads using Pilon v1.22 (10) until no further errors were detected.

Assembly quality was assessed with BUSCO 4.1.4 (11). This tool works on a database of orthologous genes and gives the number of complete, fragmented, and duplicated genes. PGAP v6.10 annotation (12) identified 3,674 and 3,745 coding sequences and 118 and 123 RNA sequences (86 and 91 transfer RNAs, 27 complete ribosomal RNAs, and 5 non-coding RNAs) in MRL14-522 and MRL14-108, respectively. PHASTER (13) (PHASTEST in its new version, 14) detected one prophage in each bacterial genome. Default parameters were used unless specified.

The final assembly of MRL14-522 and MRL14-108 consists of a single circular chromosome and a prophage for each strain (Table 1).

**TABLE 1:** Genome characteristics and sequencing data of *B. velezensis* strains MRL14-522 and MRL14-108

Strain	Sequencing platform	Nanopore reads	N50 (bp)	Illumina reads	Total genes	GC content (%)	Total size (bp)	GenBank accession
MRL14-522	GridION + NextSeq500	485,440	24,129	4,078,088	3,949	46.5	3,998,688	JBWBDH000000000
MRL14-108	GridION + NextSeq500	471,099	28,338	4,883,881	4,012	46.6	4,057,033	JBWBDI000000000

FastANI v1.34 (15) was used to determine the average nucleotide identity between *B. velezensis* type strain NRRLB-41580^T^ (2) and strains MRL14-522 or MRL14-108. Scores of 98.08% and 98.07%, respectively, confirmed their assignment to *B. velezensis* (15).

## Data Availability

These strains have been deposited in the “Collection Nationale de Cultures de Microorganismes” at Institut Pasteur (Paris, France) under the reference name CNCM I-5582 for MRL14-522 and CNCM I-5581 for MRL14-108. This Whole Genome Shotgun project has been deposited at GenBank under the accession number PRJNA1433227. The accession numbers of the complete genome sequences are JBWBDH000000000 and JBWBDI000000000, for MRL14-522 and MRL14-108, respectively. The raw sequencing reads have been deposited in the Sequence Read Archive under the accession numbers SRR37502957 and SRR37502959 for strain MRL14-522 and SRR37502956 and SRR37502958 for strain MRL14-108.
